# Biological Models of the Lower Human Airways—Challenges and Special Requirements of Human 3D Barrier Models for Biomedical Research

**DOI:** 10.3390/pharmaceutics13122115

**Published:** 2021-12-08

**Authors:** Cornelia Wiese-Rischke, Rasika S. Murkar, Heike Walles

**Affiliations:** 1 University Clinic for Cardio and Thoracic Surgery, Otto-von-Guericke-University Magdeburg, Leipziger Str. 44, 39120 Magdeburg, Germany; cornelia.wiese-rischke@med.ovgu.de; 2 Core Facility Tissue Engineering, Institute of Chemistry, Otto-von-Guericke-University Magdeburg, Pfaelzer Str. 2, 39106 Magdeburg, Germany; rasika.murkar@ovgu.de

**Keywords:** cell line, primary airway cells, complex barrier composition, iPS cell, organoids, tissue engineering, TEER measurement, live cell imaging, microfluidic chip technology

## Abstract

In our review, we want to summarize the current status of the development of airway models and their application in biomedical research. We start with the very well characterized models composed of cell lines and end with the use of organoids. An important aspect is the function of the mucus as a component of the barrier, especially for infection research. Finally, we will explain the need for a nondestructive characterization of the barrier models using TEER measurements and live cell imaging. Here, organ-on-a-chip technology offers a great opportunity for the culture of complex airway models.

## 1. Introduction

The human body has to protect itself against many environmental influences. For this purpose, it builds up very well-defined, structured, cellular barriers [1]. For example, the main function of the skin, the greatest human barrier, is to protect against unregulated loss of fluid. For this reason, the top layer of the skin, the epidermis, consists of the superficial horny layer (stratum corneum) with stratum disjunctum, the outermost layer in which the horny cells are shed, and the uncornified deep germ layer (stratum germinativum). The stratum corneum is built by dead, fused, flattened keratinocytes which are closely connected by means of desmosomes. The keratin prevents water from evaporating from the surface of the skin. The sebum, which is produced by the sebum glands, keeps the horny layer supple and water repellent [2].

**Figure 1:**
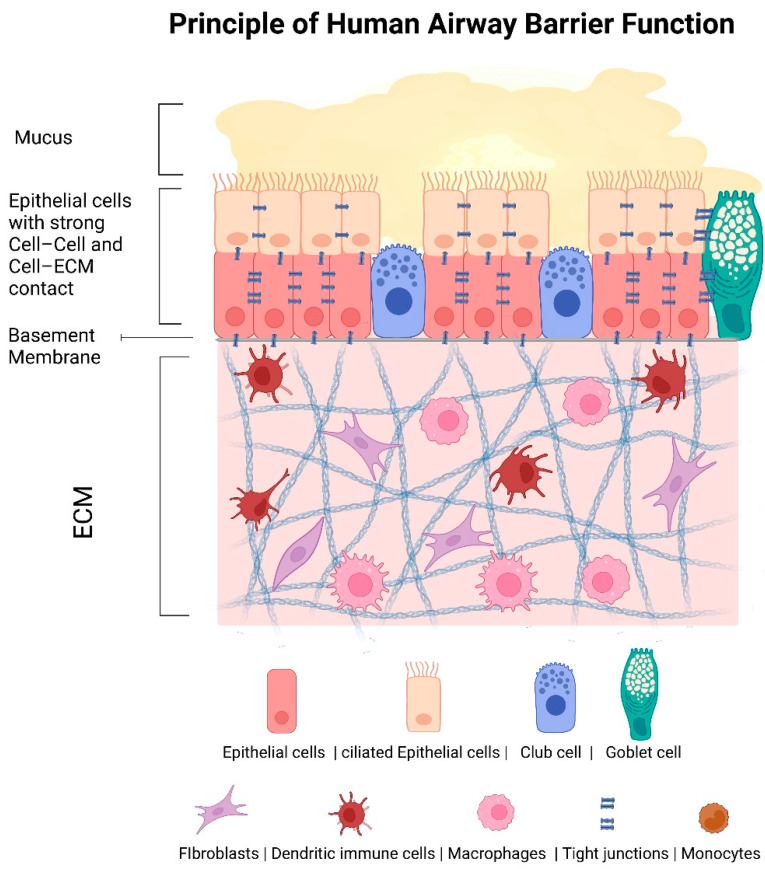
Cellular composition of the epithelial and subepithelial layers of the lower airway tract. The human lower airway tissue possesses a mucus barrier and an epithelial barrier through strong cell–cell and cell–ECM contacts. The epithelial layer contains ciliated cells that remove particles and pathogens through their cilia movement, nonciliated epithelial cells, and goblet and club cells. The subepithelial layer harbors, besides fibroblasts, also different immune cells like dendritic cells, monocytes, and macrophages. ECM—extracellular matrix. This figure was created with BioRender.com (accessed on 7 December 2021).

Another important barrier of the human body is the respiratory tract. The main function is to protect against infections of the microorganisms, which are in the air we breathe. For this reason, the airways are constructed very differently than the skin. The top layer is a layer of mucus that is formed by the cells and is permanently renewed. In addition, there are cilia-bearing cells that ensure the removal of particles and microorganisms from the human body through the coordinated movement of the cilia [3] (Figure 1).

Biomedical and pharmaceutical research has changed significantly over the past few decades. The reason for this is that the new drugs, for example biologicals and therapies, are tailored very specifically to patients or patient groups [4]. The main driver for this was the progress in sequencing technology and the associated identification of disease relevant biomarkers [5].

With this progress, the need for tissue, or disease specific markers, has grown and the discrepancy between the data from animal experiments and disease relevant mechanisms in the patient has become increasingly clear [6].

**Figure 2:**
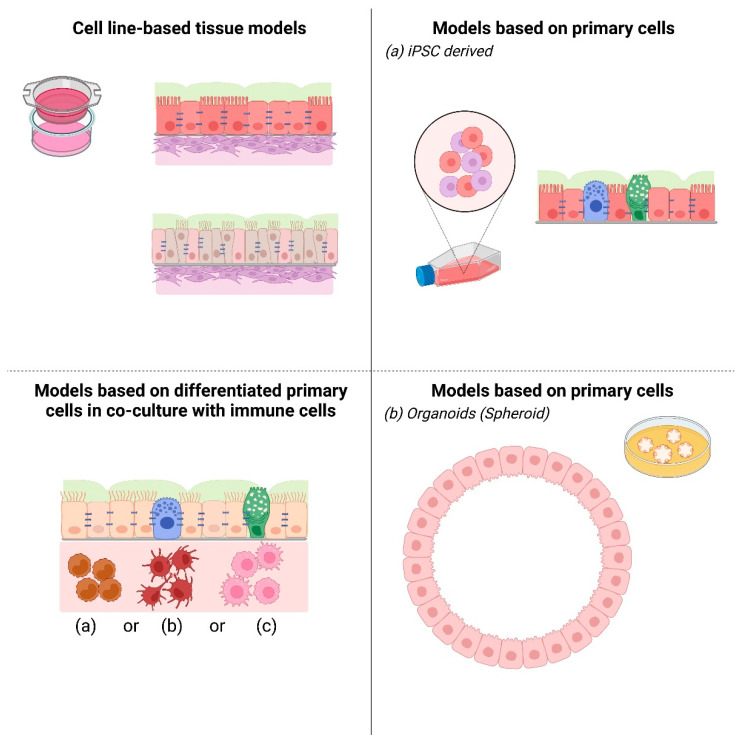
Schematic overview of 3D airway tissue model systems. This tabular scheme summarizes available 3D airway tissue models: (1) upper left: cell line-based tissue model, (2) lower left: differentiated primary cell-based co-culture models with (a) monocytes, (b) dendritic cells, or (c) macrophages, (3) right: Primary cell-based models as (a) iPS-derived model and (b) organoid culture-derived model. This figure was created with BioRender.com (accessed on 7 December 2021).

For this reason, more and more scientific teams have focused on using methods of tissue engineering or modern cell biology [7] to develop human tissue models that are suitable for the investigation of defined mechanisms. To generate these airway tissue models, cell lines, differentiated primary airway epithelial cells, induced pluripotent stem cells, and organoids were applied (Figure 2).

The research interests include the understanding of which components of the specific tissue take on which barrier function, how microorganisms act to overcome these human barriers, or which dysregulations lead to the development of diseases such as tumors. The resulting data are now used to develop targeted new therapies for specific patient groups. Numerous publications offer data generated with different cell lines and cell types generated either from cancer or immortalized cells [8,9,10,11,12]. These cell lines are extensively characterized regarding their ability to secret mucus or surfactant, their ciliogenesis, the expressed transporter proteins, and active enzymatic metabolism. However, the established two-dimensional (2D) culture of these cell lines on plastic surfaces allows less meaningful results, especially regarding the transepithelial resistance (TEER), which is built by cell–cell and cell–extracellular matrix contacts and reaches values higher than 1000 Ω/cm^2^ in vitro [13].

Due to this fact, we focus our article on three-dimensional (3D) models of the airway tissue and the influence of culture conditions as air–liquid cultures, organoids, and microfluidic chip technologies. Another focus is the relevance of specific functional cellular or tissue components as mucus or cilia and their role in infection mechanisms [14]. The scientific question we are asking is, “Can the mechanism of lung disease be investigated with such tissue models?” Further, we will provide in this review an overview on methods and technologies to create and characterize lower airway tissue models as well as the still existing challenges. Additionally, we will demonstrate the influence and importance of the mucus layer in airway models for infection studies in our own experiments.

## 2. Cell Line-Based 3D Airway Tissue Models

Different 3D cultivation methods have been developed to generate 3D airway tissue models that possess properties of the native tracheobronchial epithelium. In general, the cells can be cultivated on a scaffold and are either covered with medium during the entire cultivation time (submerged or liquid-covered), or they are exposed to air as so-called air-liquid-interface (ALI) cultivation. Due to their particular relevance, we will focus on the application of Calu-3 cells and A549 cells.

Calu-3 cells grown in submerged culture were found to exist as a monolayer, whereas ALI cultivation resulted in a pseudostratified layer of more columnar cells viewed, e.g., by trans-electron microscopy [10,12]. Desmosomes, adherence, and the presence of more or less tight junctions were observed with both cultivation methods.

Calu-3 cells are derived from a pulmonary adenocarcinoma of a 25-year-old Caucasian male in 1975 [15]. These cells intercellularly retain desmosomes, adherence junctions, and tight junctions, which were demonstrated by electron microscopy and immunofluorescence staining against desmoplakin, E-cadherin, and zonula occludens 1 (ZO-1), respectively [8,16]. At confluence, the cells show polarization typical for epithelial cells [8]. They retain constant properties over repeated passages [17]. Consistent with a serous cell phenotype, Calu-3 cells possess high levels of cystic fibrosis transmembrane conductance regulator (CFTR) and cAMP-dependent Cl-secretion [8]. They also produce secretory component, serine leukoprotease inhibitor (SLP1), lysozyme, and weakly lactoferrin [18]. Although the cells have properties of serous cells, they also express muc1 and muc2, which are found in surface goblet cells and gland mucous cells of native epithelium [18]. Therefore, Calu-3 cells reflect the properties of human bronchial submucosal glands.

In our 3D airway tissue model containing Calu-3 cells, we additionally included primary fibroblasts. Fibroblasts represent an essential part of the native peribronchial connective tissue and fulfill versatile functions [19]. A normal activated fibroblast can proliferate and migrate. These cells show contractility and secretion of growth factors and cytokines. They can further produce and remodel extracellular matrix (ECM) [20]. These factors mediate the interactions between epithelial cells and fibroblasts, which in turn regulate normal tissue development and homeostasis [21]. Fibroblasts play also an important role in several diseases such as cancer [22]. The signals from the ECM, in particular, can influence the apical–basal polarization of epithelial cells via the integrin signaling [23]. In case of Calu-3 cells, defective integrin signaling has been shown, which negatively affected their polarity in 3D culture models [24]. Fibroblast-derived signals like the hepatocytes growth factor (HGF) and the ECM were shown to restore the polarity of Calu-3 cells [24]. Both the HGF and the ECM were also described to be crucial for bronchial epithelial cell growth [21].

To establish our new 3D airway model consisting of Calu-3 cells and primary fibroblasts, we co-cultivated both cell types on a biological collagen scaffold derived from the submucosa of the porcine jejunum [19,25]. Under submerged culture conditions, a single layer of Calu-3 cells formed within 14 days (Figure S1A). In ALI cultures, a multilayered epithelium was observed (Figure S1B). The fibroblasts migrated into the scaffold under both culture conditions. In this 3D co-culture model, adherence (E-cadherin) and tight junctions (ZO-1) were present (Figure S1A,C), which suggests that an epithelial barrier was formed. MUC5AC protein and acidic mucins, which are also produced by surface goblet cells and bronchial submucosa glands, were mainly found in ALI cultures, whereas submerged cultures showed only marginal mucin production (Figure S1A,C). Thereby, this 3D co-culture model shows somewhat comparable properties with 3D Calu-3 mono-culture submerged and ALI models [10,12].

The ability to form cilia in 3D Calu-3 airway models seems not to be an intrinsic feature. Taking into account that Calu-3 cells are derived from bronchial submucosal glands, ciliogenesis in Calu-3 cell cultures would not be primarily expected [9]. Accordingly, only a few investigators observed by scanning electron microscope cilia in submerged 3D Calu-3 models in cells with passage numbers 20–40 [26,27]. Others saw approximately 30% of ciliated cells under ALI conditions and shorter and thicker cilia under submerged culture conditions [28]. Authors who looked specifically at ciliary function in different 3D airway models found that 3D ALI Calu-3 models did not display any cilia and, therefore, no ciliary beating [29]. In our co-culture 3D model, we also could not find cilia formation. In many cases, microvilli were observed with a tendency for more microvilli to form under ALI conditions [8,10,12,30]. Since microvilli are not motile, they do not participate in mucociliary clearance like cilia do. The discrepancies found in the literature were discussed as being due to variations in growth conditions and cell passage number [10], which might be reasonable and does not exclude possible other causes.

The integrity of the epithelial barrier can be tested using the transepithelial electrical resistance (TEER) measurement. In the literature, again, inconsistencies concerning the measured TEER value are reported. The TEER values in 3D Calu-3 ALI cultures vary, for example, between 100 Ω/cm^2^ [8] and 300 Ω/cm^2^ after 5 days [10], and ~400 Ω/cm^2^ after 10–14 days of ALI culture [9], and even 200–700 Ω/cm^2^ [12]. By comparison, under submerged culture conditions, higher TEER values with approximately 1000 Ω/cm^2^ [10] or 400–1700 Ω/cm^2^ [12], respectively, were determined. It also seems that co-cultivation of Calu-3 cells with fibroblasts in 3D ALI models can influence the TEER value. TEER measurements after 14 days of co-cultivation showed earlier and greater increases (~200 Ω/cm^2^) than the culture of Calu-3 cells alone (~130 Ω/cm^2^), suggesting that culture together with fibroblasts facilitated an earlier confluent-differentiated state of Calu-3 cells [11]. Several further factors may affect the TEER measurement, including temperature, passage number, cell culture medium, culture period, cell culture method, TEER-related mechanoelectronics, and individual handling, to name a few [13]. Therefore, it is difficult, if not impossible, to reliably compare the reports with each other. Furthermore, the main question of which culture conditions and measurement protocols provide TEER values that resemble the in vivo situation most arises. Reference values from human bronchial/tracheal epithelium are not available, but experiments in the 1990s with rabbit bronchial epithelium ex vivo determined a steady state electrical resistance of 266 ± 97 Ω/cm^2^ [31]. This range of TEER values is quite comparable with the reported values in 3D ALI Calu-3 models. Higher TEER measurements, however, appear to less reflect physiological values. In fact, the measurement of TEER alone may not be enough to make a statement about the barrier function. In addition, histological and structural analyses are needed to confirm the functional barrier [32]. Since TEER values contain the electrical resistance of the transcellular (membrane permeability) and paracellular (tight junction) pathways, changes in the first one may also have an impact on the TEER values [33]. Therefore, the membrane permeability is another additional parameter characterizing the epithelial barrier function.

A549 cells are derived from an adenocarcinoma of a 58-year old Caucasian male [34]. These cells retain characteristics comparable to the Calu-3 cell line regarding the cell–cell/cell–matrix contact. They express desmosomes, adherence junctions, and tight junctions and form in contrast to Calu-3 cell lamellar bodies. These function units were demonstrated by electron microscopy, immunofluorescence, and by quantitative polymerase chain reaction (qPCR) against, E-cadherin, ZO-1, claudins 1–3, and 5, respectively, whereas claudin 4 could not be detected [35].

They retain constant properties over repeated passages and are the most often applied human lung epithelial cell line in pharmacological and biomedical studies [34,35]. One reason is their ability of epithelial–mesenchymal transition (EMT) induction [36], an important mechanism in tumor research. Another scientifically very interesting aspect of this cell line is their functional characteristic of human alveolar type 2 (AT2) pneumocytes [34,35]. In detail, this includes the presence of the essential membrane mucin component, MUC1 [37], the Ren inducible secretion of mucin MUC5AC [37], the expression of alveolar transporter [38], and the expression of enzymes essential for the extracellular production of adenosine triphosphate [ATP], the enzymes alkaline phosphatase (ALP), and adenylate kinase (ADK) [39].

In 2D culture on plastic surfaces, A549 cells show a very low TEER, demonstrating a reduced epithelial barrier integrity function. TEER values monitored using “chopstick” electrode of 28 ± 4 Ω/cm^2^ on day 9 [40], with little change over the culture period, 50 ± 2 Ω/cm^2^ on day 3 [41], or 55 ± 5 Ω/cm^2^ on day 7 and stable until day 10 [41] were reported.

In comparison with the published data of the Calu-3 cell experiments, it can be summarized that the A549 cells develop significantly fewer tight junctions on plastic surfaces despite the exposure of the relevant proteins [35]. It is interesting in this context, that the expression of these proteins can be increased by culture on hydrogels [37].

For this reason, our airway models are established on a collagenous carrier structure and in co-culture with fibroblasts [19,25,36]. As described above, we have also established a co-culture model of A549 cells and primary human fibroblasts on a biological matrix. The main difference between the Calu-3 cell and A549 cell models is that the epithelial monolayer forms a much thinner mucus layer [25]. The A549 cell model, therefore, instead represents the physiological structure of the distal respiratory tract.

To establish a bacterial infection model, the collagenous scaffold was populated with 1 × 10^5^ primary fibroblasts per cell crown and one day later with 1.5 × 10^5^ A549 cells or Calu-3 cells. After 12 days of ALI cultivation, the medium was changed, the models were sucked dry and two million or four million staphylococci were added. The histological stainings showed that both models had a physiological histology [42].

However, the lysis of the cells during infection also destroyed the mucus. In addition, the bacteria were able to penetrate much deeper into the A549 cell models than into the Calu-3 cell models, which suggests that the mucus also represents a barrier to bacterial penetration in the in vitro models for a defined period of time. The fibroblasts immigrated well into the scaffold, and the epithelial cells had produced mucus. It was noticeable that the Calu-3 cell models produced significantly more mucus than the A549 cell models. TEER measurements were not possible with the established chopstick technology for 2D culture in 3D models. Therefore, in two master project theses, we developed a TEER value measuring device that enabled the characterization of the 3D models [42].

Interestingly, the barriers of the A459 co-culture models were significantly higher and more comparable with the physiological values.

## 3. Primary Cells Derived 3D Lower Airway Models

### 3.1. In Vitro 3D Cultures of Biopsies

An alternative to the above described cell lines are primary human airway epithelial cells, which can be isolated from healthy and diseased donors [21]. Certainly, this cell type has not established itself for the construction of airway tissue models, as the cells only have a very low proliferation capacity in vitro [21]. On the other hand, several research groups and companies have established diverse primary 3D airway models based on cells either isolated by bronchial brushings [43] or clinical specimens following thoracic surgery [44]. All these tissue models consist of a mixture of ciliated, secretory, and basal cells. These 3D cultures build an increased, pseudostratified epithelial layer and express the physiologically MUC5AC protein [43,44]. The advantage of these models is that they are commercially available from several suppliers. They are characterized regarding the epithelial origin by immune histology, sex, race, or disease of the donor [45]. These defined culture conditions enable extensive studies to be carried out on diseases such as chronic obstructive pulmonary disease (COPD) [46] or the influence of age and gender [47] on active ingredients of the (target) therapies [48]. The disadvantage of the primary airway models is the high variation of TEER values between 300 and 800 Ω/cm^2^ depending on the donor, passage of the primary cells [45], and the cell culture medium [32]. Several studies underline that the cell culture medium influences the ciliary beating frequency [32] and other cell functions. These commercially available airway tissue models have a high purchase price, but they are very successfully applied in drug development against viral or bacterial infection or in developing lung disease models [32,35,36,37,38,39,40,41,42,43,44,45,46,47,48].

### 3.2. iPS Differentiated Cells, Human Pluripotent Stem Cells

Alternative cell sources to generate 3D airway tissue models could be differentiated induced pluripotent stem cells (iPS) or differentiated human pluripotent stem cells (hPSC). The advantage of these cell sources is that the cells could be generated individually from each person, a prerequisite to establish advanced healthy and diseased tissue models.

One requirement for the differentiation of these pluripotent cell types is the knowledge about the signaling pathways involved in the stepwise differentiation processes. The first differentiation protocols were published from Wong and colleagues in 2012 [49]. Since 2017, several groups have published protocols to differentiate lung epithelial cells from iPS cells [50,51,52,53].

But despite all these successes, the iPS cell culture is not yet suitable for the production of functional 3D airway models, because it is still unclear whether iPS cell-derived airway epithelial cells are mature. A functional airway model must be at a minimum composed of ciliated, goblet, and basal cells. For specific scientific questions, the presence of Clara or neuroendocrine cells are necessary. Additionally, a physiological microenvironment is needed to develop functional 3D airway models.

All these missing requirements led to the development of the organoid technology in the field of 3D tissue model engineering.

### 3.3. Organoids

To overcome the above-mentioned drawbacks, methods of 3D culture technologies in collagenous microenvironments have been developed. In 1993, Benali and colleagues cultured epithelial cells isolated from nasal polyps in a 3D collagenous microenvironment. After 12 days, these cells formed self-organized tubular structures with a lumen, which was surrounded by polarized ciliated and secretory cells [54]. Based on these experiments and results, “organoids are defined as stem-cell-derived, multicellular cell systems. The cells have the potential for self-organization into differentiated, functional cell types that resemble in vivo counterparts and key features of the organ [54,55]”.

Organoids can be created based on stem-cell-containing biopsies, as described above or on differentiated human iPSCs [56]. The addition of organ specific cytokines/growth factors induce airway specific differentiation pathways in 3D spheroids cultured in a 3D extracellular microenvironment [57].

The advantage of the organoids is that they have a very high long-term self-renewing capacity; therefore, repeated isolation becomes unnecessary. The organoid technology enables the generation of airway tissue and diseases models (e.g., cystic fibrosis [58]) on genetically defined backgrounds. Anderson and colleagues published in 2021 [58] a very efficient screening based on cystic fibrosis organoids. The cells were isolated from brush biopsies of patients. A functional read out in cystic fibrosis is the swelling of the tissue models which should be influenced by drugs. The team developed a functional swelling assay including an automated measurement and analysis. This new technology enables for the first time, a parallel screening of different drugs on tissue of diverse patients. Furthermore, the individual effectiveness of monotherapies could be compared with combination therapies. Based on the comparison of the organoid generated in vitro data and the clinical responses of the patients, Anderson and colleagues were able to identify an in vitro biomarker, which can be used in the future to determine which patient will benefit from which therapy. Other teams used the PSC-derived lung organoids, which form branching airway and alveolar structures [59], to study viral infections, including the human parainfluenza virus type 3 (HPIV3), the respiratory syncytial virus (RSV), and the measles virus. Interestingly, the infection of these different viruses induced physiological morphological changes, as detachment of infected cells or syncytium formation [60]. These data underline the physiological response of the lung airway models, but they also demonstrate the challenges using 3D cell culture models for infection studies. The organoids have a lumen without access to the air; organoids are closed spheres. Microorganisms, such as viruses or bacteria do not have the first contact with the epithelial layer or the mucus. After the published infection studies, morphology changes have been demonstrated in endpoint studies, but it is not an easy applicable readout, and no data regarding the reproducibility are published so far. Other technologies to characterize the barrier function as TEER measurements cannot be applied. The culture in Matrigel^®^ and all the necessary additives to induce differentiation are very expensive and have a high batch to batch variation.

To overcome these limitations, researchers started generating tissue specific organoids to use for the seeding of either ALI culture in Transwells [61] or 3D scaffolds [62]. Lamers and colleagues have published SARS-CoV-2 infection studies in this 2D Transwell set up [61]. However, these technologies do not allow for the co-culture of immune cells, which play an important role during infection.

When 3D microporous scaffolds are seeded with organoids, they differentiate into secretory lineages [62], an interesting technology for the generation of autologous implants of the airways.

## 4. Challenges

### 4.1. Determine the Barrier Function by Measuring the Electrical Resistance (TEER)

As described above in chapter 2 in much detail, TEER measurement with the available technologies is very dependent on the experimental device, the person who is measuring, and parameters such as temperature or cell culture medium. In addition, up to now, TEER measurement has generally only been used as endpoint measurements. Since according to the measuring principle, the integrity of the tight junction in epithelial cell culture models can be measured quantitatively [63], it is an ideal technology to control the development of the epithelial barrier over culture time and to use it as an noninvasive method during drug testing. Several groups have developed new devices and technologies based on measuring Ohmic resistance (TEER) or impedance across a wide spectrum of frequencies [13]. Time-resolved impedance spectroscopy of 3D skin tissues and wound healing over time has been demonstrated [64]. Nevertheless, such studies are only published using tissue-engineered 3D barrier models in Transwell environments. With the development of advanced microfluidic 3D cell culture technologies (organ-on-chip) the integration of electrodes in the cell culture device enables the measurement of TEER during the complete tissue culture [65] of 3D tissues as intestinal or liver organoids [66].

Another strategy is to generate organoids and use this tissue specific cell composition to establish organoid-derived monolayers (ODMs) in Transwells. These ODMs can be used for physiologically relevant infection studies with *Toxoplasma gondii*, as an example. The published data underline the reproducibility of TEER measurements and the species to species variation of *Toxoplasma gondii* infection [67].

Due to the complex structure and cellular composition, which has a high variation during the anatomical course from nose to lungs, such TEER or impedance data are not available so far.

### 4.2. Involvement of Immune Cells

The airways and lungs are composed of different, highly specialized cell types that are protected by a large number of diverse immune cells of the innate and adaptive immune system as well as subgroups of dendritic, natural killer cells, regulatory T cells, and natural group 2 lymphoid cells [68,69].

In order to increase validity and relevance of cell-based airway models, immune cells would have to be integrated into the 3D models, especially for infection studies and the discovery of anti-infective drugs. All immune cells are specialized in recognizing foreign proteins from microorganisms as well as cells or nonliving particulate systems and removing them as quickly and effectively as possible.

For this reason, most of the immunocompetent airway models established to date are co-cultures with cell lines, such as the THP-1 monocyte-derived macrophages [70,71]. These co-culture airway models ensure epithelial barrier integrity and transmigration of macrophages into the tissue during the cell culture period. This is then an ideal model system to study inflammation induced by bacterial infection and survival after drug application [72].

An alternative approach is the construction of the airway model with primary cells, which were characterized with regard to the expression of immunologically relevant surface markers. After that, a co-culture can be established with well-characterized allogeneic primary macrophages [73] and dendritic cells in order to study both bacterial and fungicidal infection mechanisms [74]. The disadvantage of this technology is that complex culture conditions such as perfusion and co-culture are then necessary, and the examinations can only be carried out for a few hours.

In 2021, the first study to predict the efficacy of immune-cell-based tumor therapy applying autologous gastric cancer organoid and immune cells was published by Chakrabarti and colleagues [75]. A prerequisite for such sophisticated studies is the culture of autologous patient-derived cells. Until now, further studies regarding the co-culture of airway organoids and primary immune cells have not been published yet.

To characterize these complex co-culture models, including the migration or activities of immune cells, technologies such as the live cell imaging become increasingly important.

### 4.3. Live Cell Microscopy

In addition to measuring the transepithelial resistance, microscopy is suitable for optimal evaluation of the interaction at biological barriers. Numerous groups have proven this with microscopy. Especially with fluorescence microscopy, dynamic cellular processes and cell–microorganism interactions can be studied. One prerequisite for live cell imaging technologies is specific fluorescent labelling of target proteins, either of the cell or on the surface of viruses and bacteria. The methods for protein labelling have been optimized with regard to high specificity, protection of physiological protein function, and low cytotoxicity for the labelled cells [76].

Applying these innovative labelling methods, diverse super-resolution microscopy technologies have been developed in the last decade. Nowadays, it is possible to observe cellular components down to the nanoscale [77].

Unfortunately, these techniques cannot be used to characterize the epithelial barrier function, as the tissue models consist of several cell layers. Often 3D models have to be built up as ALI cultures. This results in a working distance that is far too large for microscopy. Three-dimensional tissue models simulating the airway and skin tissues need the interface to air as an important stimulus to develop tissue specific barrier functions [78]. On the other hand, it is an essential prerequisite for high resolution microscopy that there is no air, but liquid, between the objective and the specimen. Viktoria Zaderer and colleagues have published a very interesting technology that could enable further development of high resolution microscopy for 3D airway models as well [79]. They have demonstrated that it is possible to culture primary respiratory epithelial cells in a birch-based cellulose hydrogel. Interestingly, this hydrogel induces a faster differentiation of the epithelial cells and facilitates for the first time an upside down culture of inserts, a modified technology which allows live cell imaging of the co-cultures in Transwells. They have impressively demonstrated that they can perform multiple life cell exposures of the same airway cultures for up to two years and analyze the mucociliary clearance of their tissue models in a more physiological environment. Furthermore, this technology enables the co-culture of immune cells in the upper part of the insert, reflecting a physiological cellular arrangement in the tissue composition [79].

## 5. Conclusions

In our review, we have shown that the development of complex airway models is very important, especially for biomedical research and the development of individualized therapies. It is impressive that the existing models are used for all aspects such as infections, diseases such as tumors, or mechanisms of transport across the airway barrier. Due to the complexity of the cellular composition in the airways and the different barrier components such as mucus, cell–cell and cell–matrix contact, this development is anything but trivial for the respiratory tract and lungs. Currently, the co-culture models based on cell lines and/or primary cells are the most widely characterized. In vitro cultured biopsies can also be used very reliably in biomedical airway research. Due to the functional characterization, the iPS technology will probably only be able to be successfully implemented with the further development of the organ-on-a-chip technology. In addition to the development of the TEER measurement and the live cell microscopy, further research activities are also necessary in the field of materials research for the culture of complex models.

## Supplementary Materials

The following are available online at https://www.mdpi.com/article/10.3390/pharmaceutics13122115/s1, Figure S1: Characterization of the 3D airway tissue model composed of Calu-3 cells and primary human fibroblasts. At day one, 1 × 10^5^ human primary dermal fibroblasts were plated on cell culture inserts with a biological collagen scaffold (submucosa of decellularized porcine jejunum) and cultured in DMEM with 10% FCS. At day two, 4 × 10^5^ Calu-3 cells per insert were added and cultured together with the fibroblasts in 1:1 mixed medium (DMEM medium (high glucose) containing GlutaMAX and sodium pyruvate with 10% FCS: MEM medium containing GlutaMax supplemented with 1 mM sodium pyruvate and 10% FCS). The 3D models were cultured either submerged for 14 days (A) or cultured at ALI for up to 35 days starting from day three (B, C). The medium was changed three times a week (basally only for ALI culture). The 3D models were embedded with Tissue Tek O.C.T. compound at the respective days and cryo-sectioned at 10 µm thickness. Alcian blue staining with nuclear fast red stain was performed according to standard protocols to detect mucus proteins and to counterstain the cytoplasm and nuclei, respectively. Immunofluorescence stainings against E-cadherin (E-Cad), CD90 (fibroblasts), Muc5AC protein (mucus protein), and ZO-1 (zona occludens-1) were carried out at 14 days. The nuclei were stained with DAPI (blue). (A) In 3D submerged Calu-3/fibroblast co-culture models, a monolayered epithelium with adherens (E-Cad) and tight junctions (ZO-1) formed. (B) In 3D ALI Calu-3/fibroblast co-culture models, a multilayered epithelium formed, and the fibroblasts migrated deeply into the collagen scaffold. The mucus was stained in a light blue color. The amount of mucus increased over time. (C) The epithelial cells formed adherens (E-Cad) and tight junctions (ZO-1) and secreted mucus (Muc5AC). Scale bars are as indicated.
