# Supplementary material for: Biological Models of the Lower Human Airways—Challenges and Special Requirements of Human 3D Barrier Models for Biomedical Research
Source: Pharmaceutics. 2021 Dec 8;13(12):2115. doi: 10.3390/pharmaceutics13122115 (PMC8707984; doi:10.3390/pharmaceutics13122115)
Supplement: Supplementary file 1 [file pharmaceutics-13-02115-s001.zip › pharmaceutics-1372882-supplementary.pdf]

pharmaceutics

MDPI

Supplementary Materials: Biological Models of the Lower Human Airways—Challenges and Special Requirements of Human 3D Barrier Models for Biomedical Research

Cornelia Wiese-Rischke, Rasika S. Murkar and Heike Walles

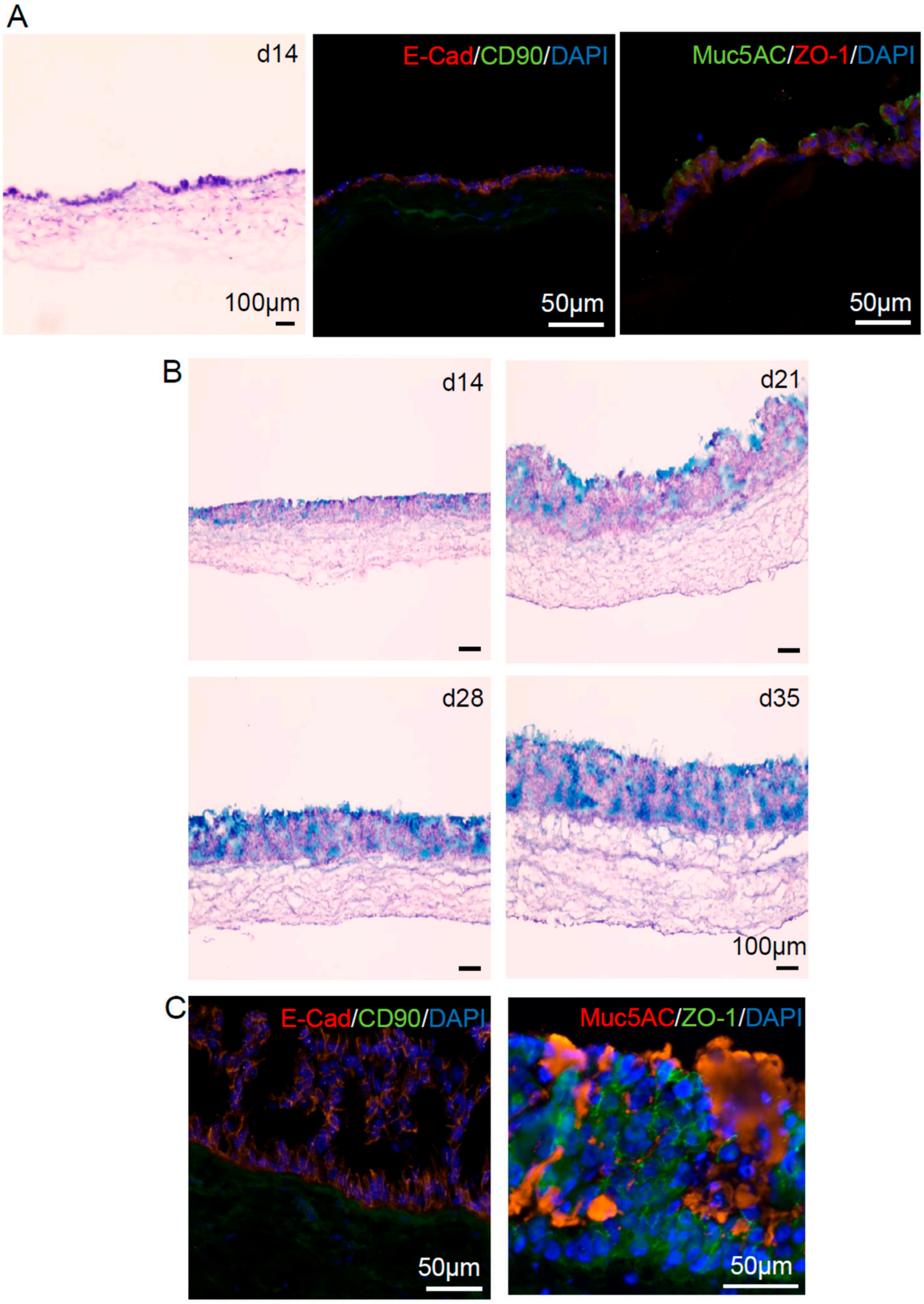

Figure S1. Characterization of the 3D airway tissue model composed of Calu-3 cells and primary human fibroblasts. At day one, 1 x 10^5 human primary dermal fibroblasts were plated on cell culture inserts with a biological collagen scaffold (submucosa of decellularized porcine jejunum) and cultured in DMEM with 10% FCS. At day two, 4 x 10^5 Calu-3 cells per insert were added and cultured together with the fibroblasts in 1:1 mixed medium (DMEM medium (high glucose) containing GlutaMAX and sodium pyruvate with 10% FCS: MEM medium containing GlutaMax supplemented with 1mM sodium pyruvate and 10% FCS). The 3D models were cultured either submerged for 14 days (A) or cultured at ALI for up to 35 days starting from day three (B, C). The medium was changed three times a week (basally only for ALI culture). The 3D models were embedded with Tissue Tek O.C.T. compound at the respective days and cryo-sectioned at 10 µm thickness. Alcian blue staining with nuclear fast red stain was performed according to standard protocols to detect mucus proteins and to counterstain the cytoplasm and nuclei, respectively. Immunofluorescence staining against E-cadherin (E-Cad), CD90 (fibroblasts), Muc5AC protein (mucus protein), and ZO-1 (zona occludens-1) were carried out at 14 days. The nuclei were stained with DAPI (blue). (A) In 3D submerged Calu-3/fibroblast co-culture models, a monolayered epithelium with adherens (E-Cad) and tight junctions (ZO-1) formed. (B) In 3D ALI Calu-3/fibroblast co-culture models, a multilayered epithelium formed, and the fibroblasts migrated deeply into the collagen scaffold. The mucus was stained in a light blue color. The amount of mucus increased over time. (C) The epithelial cells formed adherens (E-Cad) and tight junctions (ZO-1) and secreted mucus (Muc5AC). Scale bars are as indicated.
